# Covid-19 Outbreak Progression in Italian Regions: Approaching the Peak by the End of March in Northern Italy and First Week of April in Southern Italy

**DOI:** 10.3390/ijerph17093025

**Published:** 2020-04-27

**Authors:** Cosimo Distante, Prisco Piscitelli, Alessandro Miani

**Affiliations:** 1 CNR ISASI Unit of Lecce, Institute of Applied Sciences and Intelligence Systems, 73100 Lecce, Italy; 2 Euro Mediterranean Scientific Biomedical Institute (ISBEM), 72023 Mesagne (BR), Italy; priscofreedom@hotmail.com; 3 Italian Society of Environmental Medicine (SIMA), 20149 Milan, Italy; alessandro.miani@gmail.com; 4 Department of Environmental Science and Policy, University of Milan, 20122, Milan, Italy

**Keywords:** COVID-19, outbreak progression, Italian regions, peak, model

## Abstract

Epidemiological figures of the SARS-CoV-2 epidemic in Italy are higher than those observed in China. Our objective was to model the SARS-CoV-2 outbreak progression in Italian regions vs. Lombardy to assess the epidemic’s progression. Our setting was Italy, and especially Lombardy, which is experiencing a heavy burden of SARS-CoV-2 infections. The peak of new daily cases of the epidemic has been reached on the 29th, while was delayed in Central and Southern Italian regions compared to Northern ones. In our models, we estimated the basic reproduction number (R_0_), which represents the average number of people that can be infected by a person who has already acquired the infection, both by fitting the exponential growth rate of the infection across a 1-month period and also by using day-by-day assessments based on single observations. We used the susceptible–exposed–infected–removed (SEIR) compartment model to predict the spreading of the pandemic in Italy. The two methods provide an agreement of values, although the first method based on exponential fit should provide a better estimation, being computed on the entire time series. Taking into account the growth rate of the infection across a 1-month period, each infected person in Lombardy has involved 4 other people (3.6 based on data of April 23rd) compared to a value of $R_{0}=2.68$, as reported in the Chinese city of Wuhan. According to our model, Piedmont, Veneto, Emilia Romagna, Tuscany and Marche will reach an R_0_ value of up to 3.5. The R_0_ was 3.11 for Lazio and 3.14 for the Campania region, where the latter showed the highest value among the Southern Italian regions, followed by Apulia (3.11), Sicily (2.99), Abruzzo (3.0), Calabria (2.84), Basilicata (2.66), and Molise (2.6). The R_0_ value is decreased in Lombardy and the Northern regions, while it is increased in Central and Southern regions. The expected peak of the SEIR model is set at the end of March, at a national level, with Southern Italian regions reaching the peak in the first days of April. Regarding the strengths and limitations of this study, our model is based on assumptions that might not exactly correspond to the evolution of the epidemic. What we know about the SARS-CoV-2 epidemic is based on Chinese data that seems to be different than those from Italy; Lombardy is experiencing an evolution of the epidemic that seems unique inside Italy and Europe, probably due to demographic and environmental factors.

## 1. Epidemiological Figures

According to the Italian National Institute of Health (ISS), by March 29th in Italy there were more than 101,000 people who tested positive for SARS-CoV-2 since the beginning of the epidemic (75,500 currently positive and 14,600 healed) [1]. About 58% of cases are male (median age: 62 years old). Detailed epidemiological figures provided by the Italian National Institute of Health tell us that men represent the majority of cases between 50 and 89 years old (range of 55%–66%), while in the younger age groups, males and females are equally represented among people who tested positive for SARS-CoV-2. Men also represent the vast majority of deceased people in all age groups from 30 to 89 years old (range 66%–82%) [2].

Regional figures available up to 29 March show about 42,161 out of 101,000 currently positive people (42%) were detected only in Lombardy (the region of Milan), followed by Emilia Romagna (*n* = 13,531), Veneto (*n* = 8724), Piedmont (*n* = 8712), Marche (*n* = 3684), Tuscany (*n* = 4412), Liguria (*n* = 3217), Trentino Alto Adige (*n* = 3007), Lazio (*n* = 2914), Campania (*n* = 1952), Apulia (*n* = 1712), Sicily (*n* = 1501), Friuli Venezia Giulia (*n* = 1555), Abruzzo (*n* = 1345), Umbria (*n* = 1051), Sardinia (*n* = 682), Calabria (*n* = 647), Val d’Aosta (*n* = 584), Basilicata (*n* = 214), and Molise (*n* = 134). [1].

A total of 27,795 symptomatic people were hospitalized on March 30th in Italy, with Lombardy accounting for 11,815 hospital admissions (42%), followed by Emilia Romagna (*n* = 3779), Piedmont (*n* = 2985), and Veneto (*n* = 1633). Only Tuscany (*n* = 1116), Liguria (*n* = 1142), Lazio (*n* = 1079), and Marche (*n* = 998) recorded more than 600 hospital admissions at a regional level, while other regions remained lower. At the time of writing, 3981 patients remain in intensive care units, with 1330 in Lombardy (11% of hospitalized people), 452 in Piedmont (14%), 351 in Veneto (22%), 351 in Emilia Romagna (9%), 279 in Tuscany, 175 in Liguria, and 167 in Marche. With the exception of Lazio (*n* = 154) and Campania (*n* = 126), all other regions of Central and Southern Italy at the moment have less than 100 patients admitted to the ICUs of their regional healthcare systems [1].

**Figure 1:**
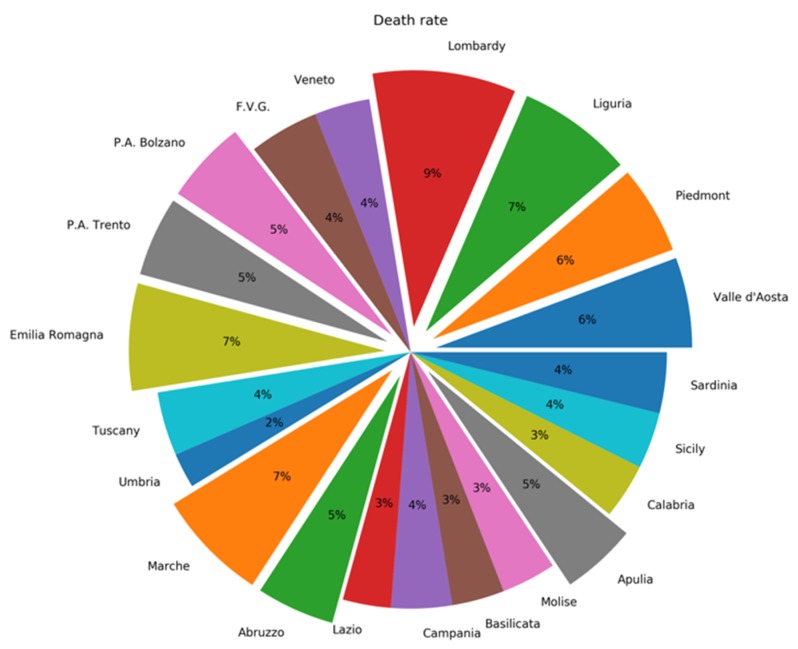
Death rate in Italy per region as of 23 April.

On 29 March, the total number of deaths at a national level was 11,951 (more than the overall 3264 deaths observed in China), with 6818 in Lombardy (57%), 1538 in Emilia Romagna (13%), 749 in Piedmont (6.2%), 417 in Marche region (3.6%), 413 in Veneto (3.5%), 397 in Liguria, 231 in Tuscany, 273 in Trentino Alto Adige, 150 in Lazio, 125 in Campania, 107 in Friuli Venezia Giulia, 102 in Abruzzo, 91 in Apulia, 76 in Sicily, and less than 50 in the other five regions (Figure 1) [1]. The lethality rate seems to increase with age and is higher in males: 0% from 0 to 29 and 0.3% between 30 and 49 years of age; 1.9% in the age group 50–59 years (0.8% in women and 2.4% in men); 6.4% from 60 to 69 years (3.5% in women and 6.9% in men); 18.5% from 70 to 79 years (11.7% in women and 19.8% in men); 26.2% from 80 to 89 years (18.9% in women and 29.2% in men); and 24.8% after 90 years of age (20.4% in women and 30.8% in men). [2].

Based on these figures, it is clear that the SARS-CoV-2 outbreak is now putting overwhelming pressure mainly on Lombardy and the Northern regions of the Po Valley (Padana Plain), but the peak of the epidemic has not yet been reached. Until now, Southern regions seemed to be less affected by SARS-CoV-2, although a huge number of people, mainly students attending universities in Northern Italy, came back from Po Valley to their families in the South in the middle of the outbreak, thus representing a potential factor able to accelerate the spread of the viral infection.

Here we present an attempt to predict the peak of the outbreak in Italy, which is expected to reach a national level by the end of March, and the different progression of the epidemic in Southern Italian regions compared to Lombardy.

## 2. Modeling the Covid-19 Outbreak Progression in Southern Italian Regions vs. Lombardy

The basic reproduction number (R_0_) is an indicator of the average number of people that can be infected by a person who has already acquired the infection. R_0_ is a metric of how contagious the disease is. Its correct estimation is extremely important for epidemiologists, especially when facing new diseases like SARS-CoV-2. R_0_ can be computed in different ways. In our models, we estimated the basic reproduction number (R_0_) both by fitting the exponential growth rate of the infection across a 1-month period and also by using a day-by-day assessment based on single observations [3]. This study makes use of the susceptible–exposed–infected–removed (SEIR) compartment model [4] to predict the spread of the pandemic in Italy. Our efforts could be helpful in the adoption of all the possible preventive measures, and to study the epidemic’s progression across Southern regions as opposed to the national trend. This metric can be biased by the optimal estimation of the basic reproductive number R_0_. It must be said that R_0_ is important if correlated with weather conditions, and that the reproductive index is reduced as the air temperature and relative humidity increases [5], according to the formula:

*R* = 3.968 − 0.0383 *Temperature* − 0.0224 *RelativeHumidity*

This means that the transmission of SARS-CoV-2 could decrease with the warmer season, and that some specific figures of the outbreak in Lombardy and Po Valley can be explained by taking into account climatic variables.

### 2.1. Modeling the Basic Reproductive Number R_0_ Exponential Framework Estimation

The exponential estimation is based on the work of Wu et al. [6], which was based on that of Zhao et al. [7], where the epidemic curve obeyed an exponential growth. As of the date of this study (23 April 2020), the epidemic growth was still near exponential, and the fitted model had many inlier data points.

**Figure 2:**
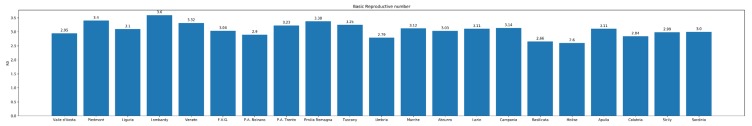
Basic reproductive index computed with the exponential fitting method along the entire time series (1-month period).

The method is based on a non-linear least square framework for intrinsic growth estimation γ, in order to obtain $R_{0}=\frac{1}{M\left(-\gamma \right)},$ with *M* being the Laplace transforming the probability distribution of the serial interval $T_{g}$ of the infection. The $R_{0}$ estimation is obtained with 100% susceptibility for SARS-CoV-2 at the early stage in Wuhan, as reported in [8]. In Figure 2, the $R_{0}$ number estimates are computed for the Southern Italian regions and for the initial outbreak region (Lombardy). According to our model, in Lombardy, each infected person has involved 4 people (3.6). The R_0_ lowers to 3.14 for Campania, which shows the highest value among the Southern Italian regions, followed by Apulia (3.11), Sicily (2.99), Abruzzo (3.0), Calabria (2.84), Molise (2.6), and Basilicata (2.66).

### 2.2. Daily Basis Estimation of the Reproductive Number

R_0_ is an average value, but it can also be computed day-by-day to monitor the transmission of the infection. Being an average value, it can be skewed by super-spreader events. A super-spreader is when an infected individual infects an unexpectedly large number of people. In Italy, this event can be also generated not necessarily by an individual, but from the perturbation of a susceptible population, as occurred in Apulia and Sicily with an uncontrolled large group of people coming from areas experiencing an outbreak. For a super-spreader, such events are not necessarily a bad sign as they can indicate that fewer people are perpetuating an epidemic. Super-spreaders may also be easier to identify and contain, since their symptoms are likely to be more severe. In short, R_0_ is a moving target. Tracking every case and the transmission of a disease is extremely difficult, so the estimation of R_0_ is a complex and challenging issue; estimates often change as new data becomes available.

If we define the *Y*(*t*) as the number of infected people with symptoms at time *t*, the exponential growth rate is λ=ln(Y(t)/t).

Let us consider $T_{g}=7.5$ as the generation time (i.e., the serial interval) and $T_{l}=5.2$ as the latent or incubation time (values taken from [6]). The infectious time $T_{i}=T_{g}-T_{l}$, and the ratio of exposed period to generation time is $\rho =T_{l}/T_{g}$. The basic reproductive number can be approximated to:

$$R_{0}=1+\lambda T_{g}+\rho \left(1-\rho \right){\left(\lambda T_{g}\right)}^{2}.$$

In order to estimate $R_{0}$, it is important to find λ and then the number of infected people:

Y(t)=suspect×p+confirmed

“suspect” corresponds to the number of individuals screened with the test which have been confirmed.

**Figure 3:**
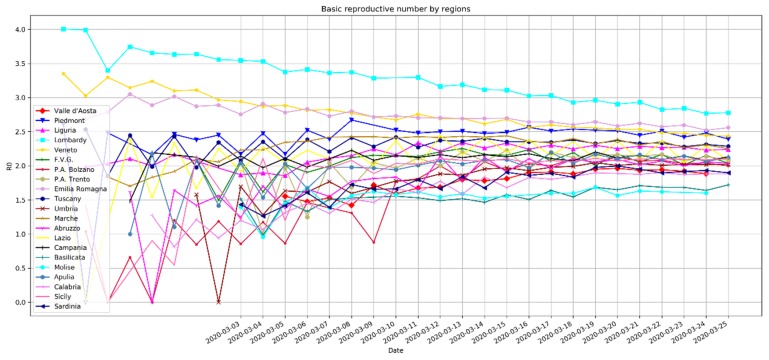
Basic reproductive index computed on a daily basis with the second method investigated in this letter.

**Figure 4:**
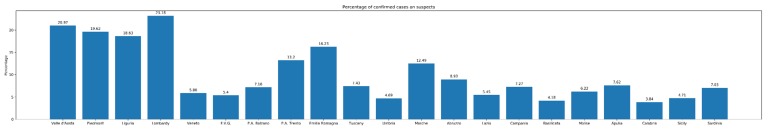
Percentage of suspects confirmed as of 23 Apri. Between 28 and 29 March, Lombardy increased by 10% with respect to the previous 24 h.

Figure 3 and Table 1 show the estimated $R_{0}$ values, computed on a daily basis for the Italian regions and for the initial outbreak in Lombardy, where about 40 cases were confirmed out of 100 suspects (Figure 4). The two methods provide an agreement of values, although the first method based on an exponential fit should provide a better estimation, having been computed on the entire time series. From Figure 3, it becomes an important aspect with respect to the Wuhan $R_{0}=2.68$ as reported in [6].

### 2.3. Modeling Transmission in Italy

**Figure 5:**
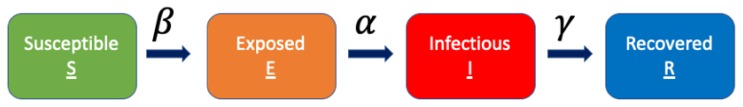
SEIR model with the four compartments and their relationships.

We used the susceptible–exposed–infectious–recovered (SEIR) model [4,7] to simulate the epidemic since it was established on January 2020. It is based on a previous model, SIR, which was based on three compartments, but since the infection has an incubation period, the compartment E (exposed) was included as shown in Figure 5. These compartments are modeled over time and capture the changes in the population. Let us say that, given N is the total population, then N = S + E + I + R, where:

“S” (susceptible): the portion of the population that does not have any vax coverage or immunity;

“E” (exposed): the portion of the population that has been infected but is in the incubation period that does not infect other individuals;

“I” (infectious): the portion of N that is infectious and may infect others, resulting in either death or recovery;

“R” (recovered): the number of infectious people who have healed and become immune.

This model captures the dynamics of these compartments over time by four ordinary differential equations. One of the most important aspects of these ordinary differential equations is equilibrium, which is achieved by setting their derivatives to 0 along time *t*. The two equilibriums are disease-free equilibrium (DFE) and endemic equilibrium (EE).

Besides equilibrium, stability is an issue correlated with the basic reproductive number, where DFE is stable if $R_{0}<1$; when $R_{0}>1$ DFE is unstable and EE is stable. The four equations are:

$$\frac{\partial S}{\partial t}=-\frac{\beta SI}{N}$$

$$\frac{\partial E}{\partial t}=-\frac{\beta SI}{N}-\alpha E$$

$$\frac{\partial I}{\partial t}=\alpha E-\gamma I$$

$$\frac{\partial R}{\partial t}=\gamma I$$

where $\beta =R_{0}/T_{i}$, $\alpha =1/T_{l}$, and $\gamma =1/T_{i}$, with $T_{i}$ and $T_{l}$ as defined above being the serial and incubation period, respectively. The contact rate β is the rate of infection from an infected individual to a susceptible contact on the unitary time step dt. The number of individuals transferred from the susceptible state to the exposed state is $\frac{\beta \cdot S\cdot I}{N}\Delta t$. The force of infection is defined as β*S(t)/N, which is the number of new infections divided by the Italian population. At the same time step, there are αE(t)Δt number of cases that are transferred from the exposed to infectious compartment, and γI(t)Δt number of cases transferred from the infectious compartment to “removed”. It is important to state that we assumed a closed population, which means the population is fixed with no births, no deaths, and no introduction of new individuals. From the above ODE system, $\frac{d}{dt}\left[S\left(t\right)+E\left(t\right)+I\left(t\right)+R\left(t\right)\right]$ = 0, which means that the population *N* is constant at any time step t: S(t)+E(t)+I(t)+R(t)=N for any t≥0. The individuals in the exposed state are infected but not yet infectious. The population is well-mixed, and the model assumes that the latent and infectious times of the pathogen are exponentially distributed. In this letter, contact rate β is changing over time as it happens in SARS-CoV-2, which increased in the early stages due to public unawareness of the disease, then decreased with government control policy measures. The contact rate follows a logistic function trend by estimating it day by day [9]:

$$\beta \left(t\right)=\frac{Ce^{-\sigma \left(t+b\right)}}{{\left(1+e^{-\sigma \left(t+b\right)}\right)}^{2}},$$

where t is the number of days after January 31st (the first found cases in Italy), σ a regularization parameter, and b the bias. A training procedure was performed on the observable data in order to find optimal (C,σ,b).

**Figure 6:**
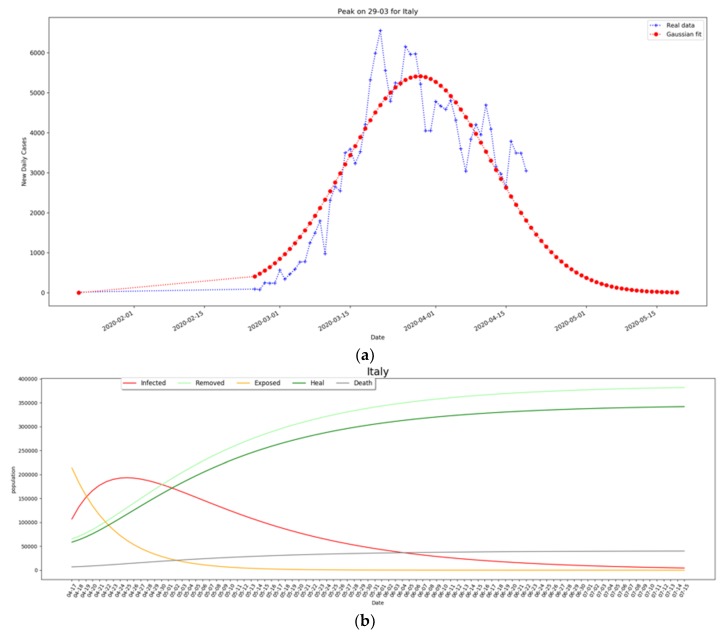
Prediction of the peak for Italy of newly active cases occurred on March 29^th^ (**a**), and exposed, infected, and the deceased shown in (**b**). An estimate of the peak of more than 180,000 active cases is shown to occur on the second half of April.

We considered with exposed people the number of twice-infected people after lockdown, which is in line with the predictions and the observed values. As shown in Figure 6a, the new daily cases peak of 29 March is shown and predicted with a Gaussian fit, while in Figure 6b (red curve), the expected peak of the SEIR model is at the second half of April at a national level. It is expected that Southern Italian regions could reach the peak later, in the second half of April. Regarding the number of the active cases peak, it must be noted that, according to [10], several cases are undocumented, so the amplitude of the peak also takes into account a small portion of undocumented cases. In a specific study carried out China before the lockdown, the author estimated that 86% of all infections were undocumented, highlighting the importance of setting up a quarantine procedure to limit the spread. As reported in [11], mobility is another important aspect for the diffusion of the virus, where they show that travel restrictions are useful in the early stage of an outbreak.

## 3. Conclusions

This paper has introduced the study of SARS-CoV-2 in Italy, by studying the evolution of the epidemic at regional level. We have experimented two different techniques to compute the basic reproduction number, one related to an estimate on a daily basis and the other on the studied period. The daily basis estimation is useful for the used compartment SEIR model to estimate the epidemic peak at national level. We showed correct prediction on the new daily cases peak occurred on March 29^th^, and gave an estimate of the active cases peak at national level. Future works will be oriented to forecast new daily cases by training deep neural networks methods, whose results will be inserted in Dinamyc SEIR model to reach better epidemic peak estimation.

## Figures and Tables

**Table 1:** Values of the analyzed regions. Given the trend, min values usually refer to the beginning of the infection.

Region	$\mathrm{Estimated}\;R_{0}$		Region	$\mathrm{Estimated}\;R_{0}$	
	min	max		min	max
Valle d’Aosta	1.57	2.11	Umbria		2.16
Piedmont	2.36	2.98	Tuscany	2.11	2.84
Lombardy	2.94	4.27	Lazio	2.10	3.11
Liguria	2.02	2.47	Abruzzo	1.29	2.36
P.A. Bolzano	0.84	2.26	Campania	1.62	2.44
P.A. Trento	1.00	2.38	Molise	1.06	1.78
Friuli.V.G.	1.74	2.52	Apulia	1.00	2.32
Veneto	2.60	3.64	Basilicata	1.19	1.73
Emilia Romagna	2.68	3.26	Calabria	0.99	2.02
Marche	1.96	2.65	Sicily	1.45	2.22
			Sardinia	1.42	2.12

## Patients Involvement

No patients were involved.
